# Distinct Innate Immune Gene Expression Profiles in Non-Melanoma Skin Cancer of Immunocompetent and Immunosuppressed Patients

**DOI:** 10.1371/journal.pone.0040754

**Published:** 2012-07-13

**Authors:** Beda Muehleisen, Shang Brian Jiang, Julie A. Gladsjo, Monika Gerber, Tissa Hata, Richard L. Gallo

**Affiliations:** 1 Division of Dermatology, University of California San Diego, La Jolla, California, United States of America; 2 Department of Dermatology, Zurich University Hospital, Zurich, Switzerland; 3 Department of Medicine, University of California San Diego, La Jolla, California, United States of America; 4 Veterans Administration San Diego Healthcare System, San Diego, California, United States of America; Ohio State University Medical Center, United States of America

## Abstract

Squamous cell carcinoma (SCC) and basal cell carcinoma (BCC) are the most frequent skin cancers in humans. An intact immune system is critical for protection against SCC since organ transplant recipients (OTR) have a 60- to 100-fold higher risk for developing these tumors. The role of the innate immune system in tumor immunosurveillance is unclear. Our aim was to determine the expression of selected innate immune genes in BCC and SCC arising in immunocompetent and OTR patients. Lesional and peri-lesional skin from 28 SCC and 19 BCC were evaluated for mRNA expression of toll-like receptors (TLR) 1–9, downstream TLR signaling molecules, and antimicrobial peptides. 11 SCC occurring in OTR patients were included in the analysis. We found that SCC but not BCC showed significantly elevated expression of TLRs 1–3, 5–8, TRIF and TRAF1. TNF was increased in SCC compared to normal skin. BCC showed increased IFNγ. hBD1, hBD2 and psoriasin mRNA and protein expression were significantly higher in SCC than in normal skin and higher than in BCC. SCC from OTR showed only an increase in hBD2 but no increase in hBD1 or psoriasin. We conclude that innate immune gene expression in SCC is distinct from normal skin and BCC. BCC shows lesser induction of innate immune genes. SCC from OTR patients have depressed expression of hBD1 and psoriasin compared to SCC from immunocompetent patients.

## Introduction

Basal cell carcinoma (BCC) and squamous cell carcinoma (SCC) are the most frequent non-melanoma skin cancers (NMSC) in humans [1]. In the U.S.A. more than 3 million cases of BCC and SCC are estimated to occur annually [2]–[3]. However, although significant progress has been made in understanding the pathogenesis of NMSC, the host immune defense mechanisms that predict patient outcome are still largely unknown. The highly increased incidence of skin cancers in immunosuppressed patients such as organ transplant recipients (OTRs) [4] has clearly shown the importance of the immune system in controlling the development of NMSC. Furthermore, because ultraviolet (UV) radiation has important immunomodulatory properties [5]–[6], its contribution as a major risk factor for NMSC is amplified.

Currently, most research on tumor immunosurveillance in NMSC, including those within the framework of cancer immunoediting [7]–[8], have focused mainly on T-cell mediated, adaptive immune mechanisms. However, there is evidence that innate immune mechanisms are also important in NMSC. Early studies by William Coley demonstrated spontaneous tumor regression after developing postsurgical infections [9], and experimental administration of *Streptococcus pyogenes* has also resulted in tumor regression [10]. Today it is well known that infectious agents can be recognized by keratinocytes and other immune cells through various genes of the innate immune system such as toll-like receptors (TLRs). Recognition of microbes results in expression of antimicrobial peptides (AMPs), cytokines and chemokines [11]–[15].

The activation of an innate immune response has diverse consequences. AMPs can kill microbes and prevent infection [13], [16]–[17]. Furthermore, AMPs can act in a manner similar to chemokines and have pro- and anti-inflammatory effects, stimulate angiogenesis and induce cell death [12], [14], [18] - effects that might be relevant also in carcinogenesis. Prior work in tumors such as oral squamous cell carcinoma showed enhanced expression of the AMPs human β-defensin 2 and psoriasin and this correlated with the clinicopathological features [19]–[20]. Furthermore, the expression of cathelicidin by NK cells has been directly shown to limit the growth rate of melanoma in mouse models [21]. More evidence for a role of the innate immune system in NMSC stems from treatment with TLR7 agonist imiquimod which is effective against superficial primary skin tumors and cutaneous metastases, including BCC, actinic keratosis and Bowen’s disease [22].

Since very little data has been published on innate immune responses in NMSC we wished to provide an overview of innate immune gene expression in a population of patients with SCCs and BCCs, focusing on TLRs, TLR-signaling intermediates, cytokines and antimicrobial peptides. Since OTRs have a significantly higher risk for SCC [4], this study also included 11 SCCs from OTRs to characterize innate immune gene expression in that high risk population. We report that NMSCs have distinct expression patterns of several genes critical to innate immunity.

## Results

### Enhanced mRNA Expression of Epidermal Differentiation Genes in SCC

**Figure 1:**
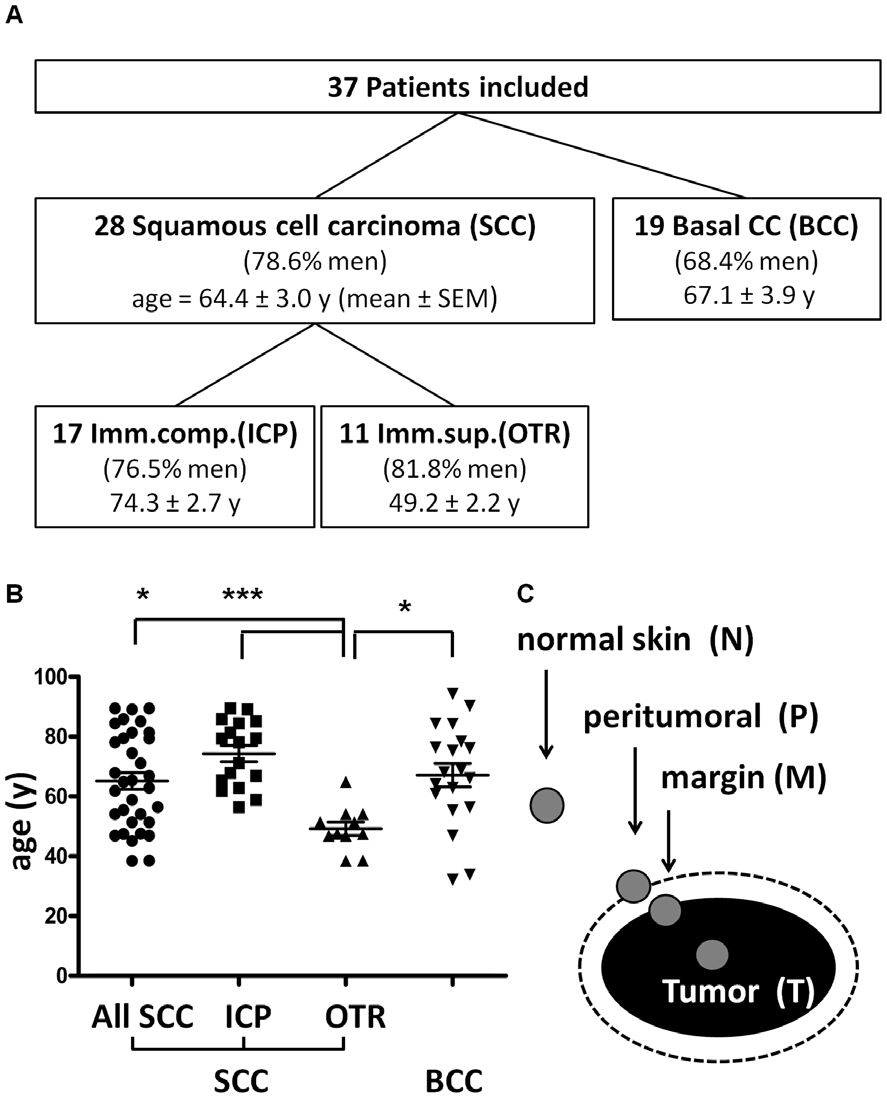
Patients enrolled and sample collection. 1A) Patients enrolled in the study, tumor diagnoses and immune status. SCC  =  squamous cell carcinoma. BCC  =  basal cell carcinoma. Imm’comp. (ICP)  =  immunocompetent patients. Imm.sup.(OTR)  =  organ transplant recipients (immunosuppressed patients). Gender (percentage male) and age (mean ± SEM) distribution in each group of patients is shown. 1 B) Age distribution in patient groups. Scatter plots including mean ± SEM are shown. *p < 0.05, ***p < 0.005 (one-way ANOVA with Bonferroni‘s post-test). 1 C) From each case, a specimen was collected from the center of the tumor (T), from the tumor margin (M) including tumor cells as well as non-tumor cells, from peritumoral tissue (P) that does not contain any tumor cells, and from normal control skin (N) in far distance from the tumor.

A total population of 37 patients was included in this study, comprising 28 patients with SCC and 19 with BCC. SCCs came from 17 immunocompetent patients and 11 that were OTR. Patient demographics in each group were similar with the exception of OTR who were generally younger (**Figure 1a,b**). Samples for analysis were taken from distant normal skin, peritumoral skin, at the tumor margin, and from central tumor tissue (See methods and **Figure 1c**).

**Figure 2:**
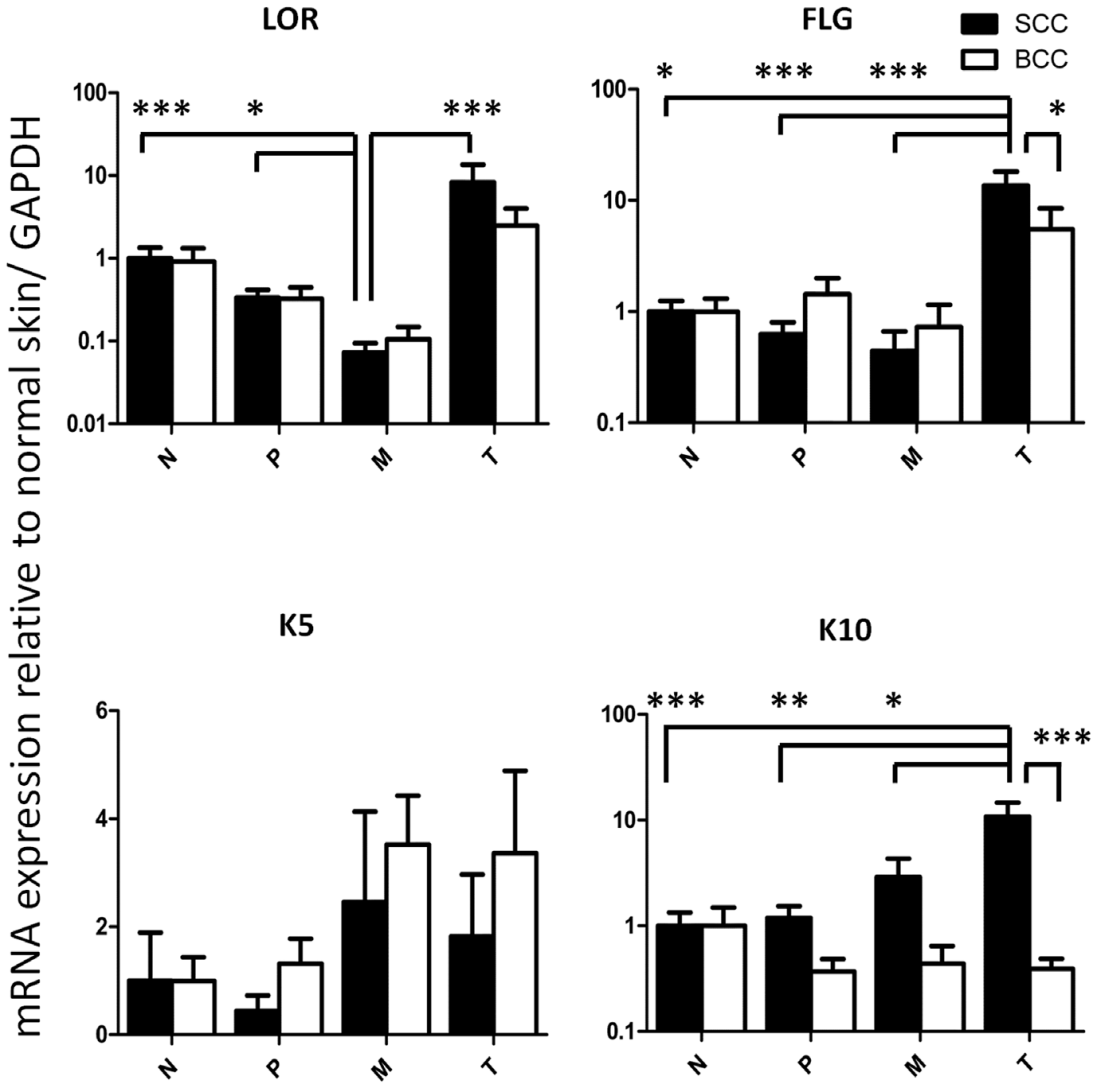
Distinct mRNA expression of differentiation genes in non-melanoma skin cancer. mRNA expression of differentiation gene loricrin (LOR), filaggrin (FLG), keratin 5 (K5) and keratin 10 (K10) in the tumor center (T), tumor margin (M), peritumoral tissue (P) and distant normal skin (N) in cutaneous squamous cell carcinoma (SCC, black bars) and basal cell carcinoma (BCC, white bars). Relative mRNA expression to glyceraldehyde 3-phosphate dehydrogenase (GAPDH) and normalized to normal skin is shown. Depicted are mean ± SEM values. *p<0.05, **p<0.01, ***p<0.001 by Kruskal-Wallis test with Dunn’s post-test for comparison between N, P, M and T and by Mann Whitney U test for comparison between SCC and BCC in each group. N = 28 SCC and 19 BCC.

Cutaneous SCC typically retains characteristics of squamous differentiation. To further characterize the specimens in our study, we measured mRNA expression of several epidermal differentiation genes. Samples within the central tumor tissue of SCC showed significantly elevated mRNA expression of filaggrin (13.60±4.52-fold (mean ± SEM), p<0.05) and keratin 10 (10.83±0.51-fold, p<0.001) compared to normal skin. Filaggrin and keratin 10 expression in the tumor center was also higher compared to the tumor margin (0.44±0.22, p<0.001; 1.56±0.51, p<0.05) and to peritumoral tissue (0.62±0.18, p<0.001; 1.19±0.34, p<0.01). Filaggrin and keratin 10 expression in the tumor center of SCC was also significantly higher than in the tumor center of BCC (5.50±2.98, p<0.05; 0.39±0.09, p<0.001). For loricrin, reduced mRNA expression in the tumor margin of SCC was observed compared to normal skin (0.073±0.021, p<0.001), the tumor center (8.29±5.29, p<0.001) and peritumoral tissue (0.33±0.08, p<0.05) (**Figure 2**).

### Enhanced TLR Expression in SCC

TLR1 mRNA expression in the SCC tumor center was 5.49±1.21-fold higher (p<0.01) than in normal skin and also significantly higher than in the tumor center of BCCs (1.82±1.21, p<0.001). TLR2 mRNA expression in the SCC tumor center was 7.90±1.76-fold higher (p<0.01) than in normal skin and also higher than in the tumor center of BCCs (1.41±0.35, p<0.001). TLR3 mRNA expression in the SCC tumor center was 8.51±2.11-fold higher (p<0.01) than in normal skin and also higher than in peritumoral tissue (1.61±0.70, p<0.05). TLR5 mRNA expression in the SCC tumor center showed the largest magnitude difference and was 30.8±22.9-fold higher (p<0.05) than in normal skin and also significantly higher than in the tumor center of BCCs (0.92±0.46, p<0.01). TLR6 mRNA expression was significantly higher in the SCC tumor center (4.48±1.44) than in the tumor margin (0.96±0.48, p<0.05). TLR7 mRNA expression in the SCC tumor center was 15.6±3.70-fold higher than in normal skin (p<0.001) and also significantly higher than in peritumoral tissue (3.37±1.35, p<0.01) and than in the tumor center of BCCs (1.98±0.53, p<0.001).

**Figure 3 pone-0040754-g003:**
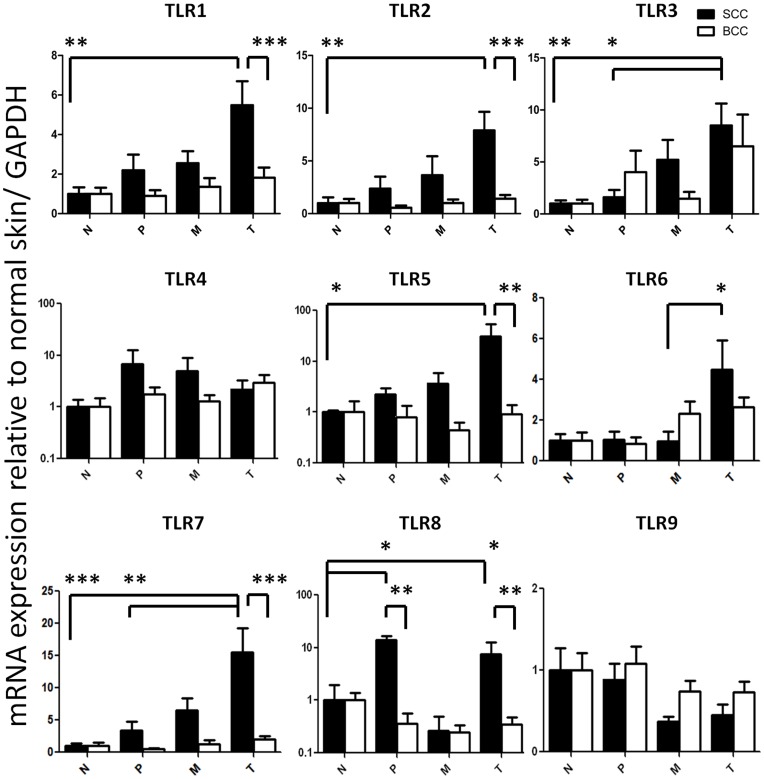
Distinct expression of toll like receptor genes in non-melanoma skin cancer. mRNA expression of toll like receptor genes 1–9 (TLR1-9) in the tumor center (T), tumor margin (M), peritumoral tissue (P) and distant normal skin (N) in cutaneous squamous cell carcinoma (SCC, black bars) and basal cell carcinoma (BCC, white bars). Relative mRNA expression to glyceraldehyde 3-phosphate dehydrogenase (GAPDH) and normalized to normal skin is shown. Depicted are mean ± SEM values. *p<0.05, **p<0.01, ***p<0.001 by Kruskal-Wallis test with Dunn’s post-test for comparison between N, P, M and T and by Mann Whitney U test for comparison between SCC and BCC in each group. N = 28 SCC and 19 BCC.

Of note, TLR8 mRNA expression was significantly higher in the peritumoral tissue of SCC (13.93±0.57) as well as in the tumor center (7.46±4.99) compared to normal skin (p<0.05 for both) and significantly higher compared to BCC peritumoral (0.36±0.21) and BCC tumor center tissue (0.35±0.14) (p<0.01 for both) (**Figure 3**).

### Enhanced TRIF, TRAF1 and TNF Expression in SCC. Enhanced TNF and IFNγ Expression in BCC

**Figure 4:**
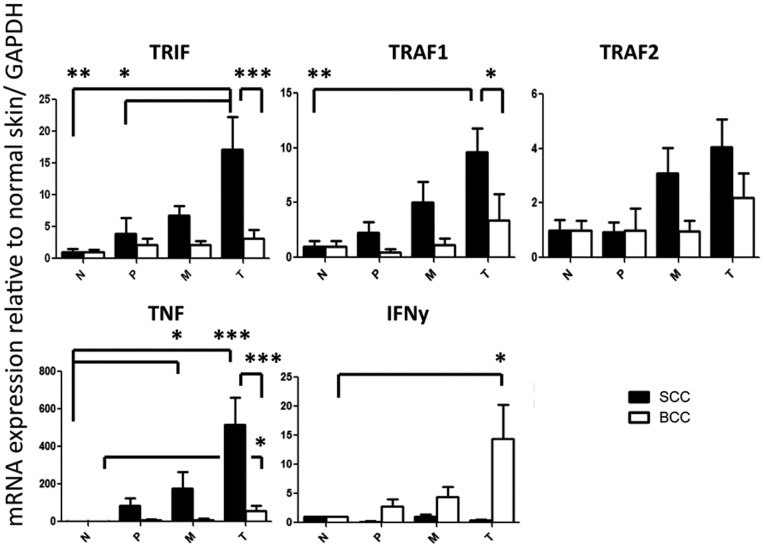
Distinct expression of innate immune receptor signaling molecules and cytokines in non-melanoma skin cancer. mRNA expression of TIR-domain-containing adaptor-inducing interferon-β (TRIF), TNF receptor-associated factors 1 and 2 (TRAF1 and 2), tumor necrosis factor (TNF) and interferon-γ (IFNγ) in the tumor center (T), tumor margin (M), peritumoral tissue (P) and distant normal skin (N) in cutaneous squamous cell carcinoma (SCC, black bars) and basal cell carcinoma (BCC, white bars). Relative mRNA expression to glyceraldehyde 3-phosphate dehydrogenase (GAPDH) and normalized to normal skin is shown. Depicted are mean ± SEM values. *p<0.05, **p<0.01, ***p<0.001 by Kruskal-Wallis test with Dunn’s post-test for comparison between N, P, M and T and by Mann Whitney U test for comparison between SCC and BCC in each group. N = 28 SCC and 19 BCC.

Consistent with the increased expression of TLRs in SCC, gene products that are either downstream of the TLR signaling cascade, or inducible by TLR activation, were also elevated in SCC. TRIF mRNA expression in the SCC tumor center was 17.11±5.14-fold higher than in normal skin (p<0.01), in peritumoral tissue (3.89±2.51, p<0.05) and also significantly higher than in the tumor center of BCCs (3.13±1.36, p<0.001). TRAF1 mRNA expression in the SCC tumor center was 9.62±2.18-fold higher than in normal skin (p<0.01) and also significantly higher than in the tumor center of BCCs (3.40±2.36, p<0.05). TNF mRNA expression in the SCC tumor center was 515±144-fold higher than in normal skin (p<0.001) and also significantly higher than in the tumor center of BCCs (55.5±29.3, p<0.001). In SCC, TNF mRNA expression was also significantly elevated in the tumor margin compared to normal skin (176.2±89.4, p<0.05). BCC showed also significantly higher TNF expression (55.5±29.3 vs. 1.02±0.55, p<0.05) and higher IFNγ expression (17.1±4.1 vs. 1.01±0.04, p<0.05) than in normal skin (**Figure 4**).

### Enhanced Expression of hBD1 and 2 and Psoriasin in SCC. BCC Shows Less Expression of CAMP, hBD1-3, RNase7 and Psoriasin than SCC

hBD1 mRNA expression in the SCC tumor center was 50.36±28.90-fold higher than in normal skin (p<0.05), in the tumor margin (0.93±0.44, p<0.05) and also significantly higher than in the tumor center of BCCs (1.08±0.45, p<0.001). In BCC hBD1 mRNA expression was significantly lower in the tumor margin than in the tumor center (0.15±0.04 vs. 1.08±0.45, p<0.05). hBD2 mRNA expression in the SCC tumor center was 4748±3934-fold higher than in normal skin (p<0.005), in the tumor margin (5.02±2.11, p<0.01) and also significantly higher than in the tumor center of BCCs (58.36±40.56, p<0.05). In BCC hBD2.

**Figure 5:**
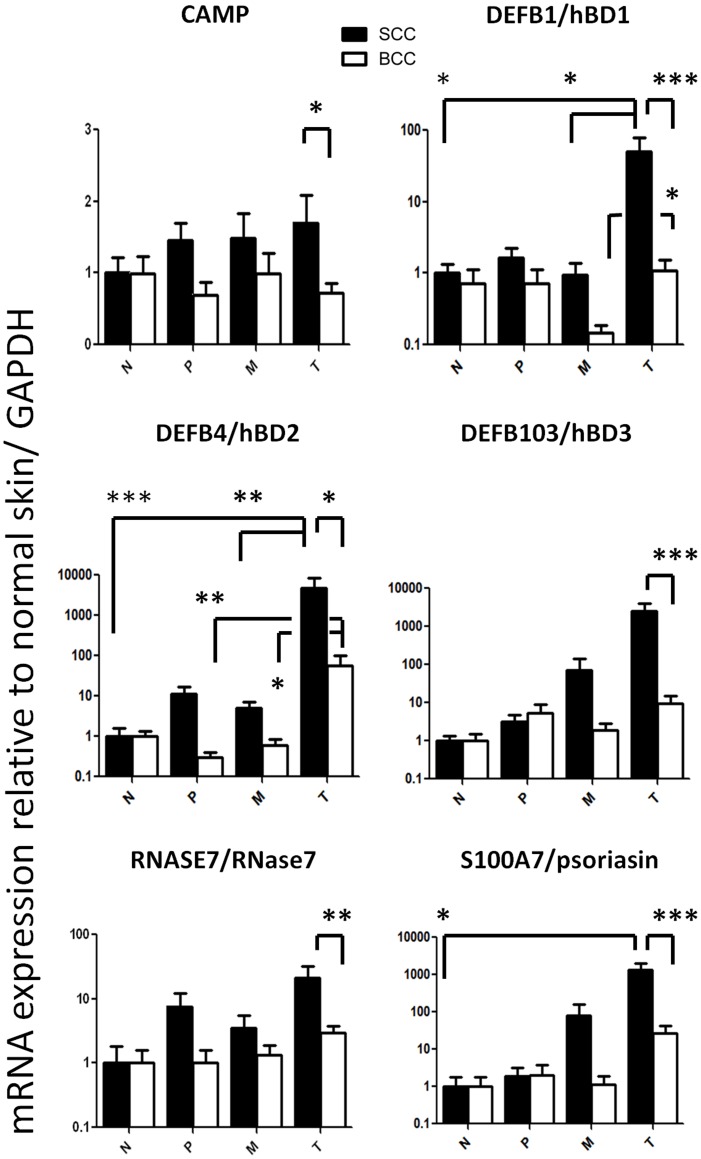
Distinct expression of antimicrobial peptides in non-melanoma skin cancer. mRNA expression of cathelicidin antimicrobial peptide (*CAMP*), human β-defensins 1–3 (hBD1–3, genes *DEFB1*, *DEFB4* and *DEFB103*), RNase 7 (*RNASE7*) and psoriasisin (S100A7) in the tumor center (T), tumor margin (M), peritumoral tissue (P) and distant normal skin (N) in cutaneous squamous cell carcinoma (SCC, black bars) and basal cell carcinoma (BCC, white bars). Relative mRNA expression to glyceraldehyde 3-phosphate dehydrogenase (GAPDH) and normalized to normal skin is shown. Depicted are mean ± SEM values. *p<0.05, **p<0.01, ***p<0.001 by Kruskal-Wallis test with Dunn’s post-test for comparison between N, P, M and T and by Mann Whitney U test for comparison between SCC and BCC in each group. N = 17 SCC from immunocompetent patients (ICP) and 19 BCC.

**Figure 6:**
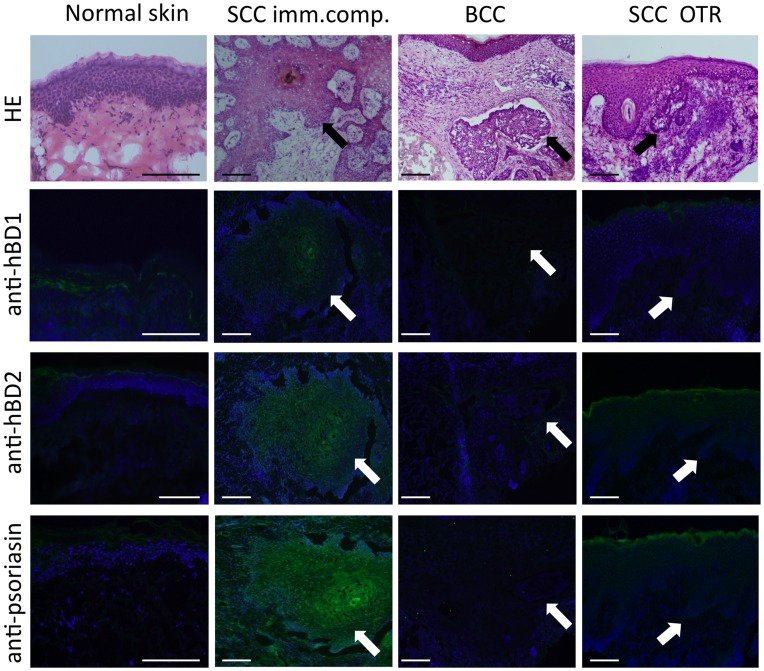
Distinct protein expression of the antimicrobial peptides hBD1, hBD2 and psoriasin in squamous cell carcinoma of immunocompetent patients and organ transplant recipients. Protein expression of the antimicrobial peptides hBD1, hBD2 and psoriasin as detected by immunofluorescence staining (green) in normal skin, basal cell carcinoma (BCC) and squamous cell carcinomas in immunocompetent patients (SCC imm.comp.) and organ transplant recipients (SCC OTR). Nuclei were visualized with 4′-6-diamidino-2-phenylindole (DAPI, blue). The top row shows hematoxylin and eosin (HE) staining of the same skin samples. Arrows point at groups of tumor cells. Bar  =  100 µm. Data are representative of five samples each.

mRNA expression was significantly higher in the tumor center (58.36±40.56) than in the tumor margin (0.61±0.26, p<0.05) or in the peritumoral tissue (0.30±0.11, p<0.01). Psoriasin(S100A7) is an abundant protein in the epidermis with much different antimicrobial activity than hBDs or cathelicidins. At protein concentrations exceeding 100 ug/ml psoriasin can partially inhibit the growth of Gram negative bacteria while hBDs and cathelicidins act at low micromolar concentrations against a broad range of bacteria, viruses and fungi [12], [23]–[26]. mRNA expression for psoriasin in the SCC tumor center was 1334±690-fold higher than in normal skin (p<0.05) and also significantly higher than in the tumor center of BCCs (27.41±14.50, p<0.001). Cathelicidin and hBD3 mRNA expression were significantly lower in the tumor center of BCC compared to SCC (0.72±0.14 vs. 1.71±0.39, p<0.05; 9.59±5.15 vs. 2497±1447, p<0.001) (**Figure 5**). In line with elevated mRNA expression, immunofluorescence staining also revealed enhanced protein expression for hBD1, hBD2 and psoriasin in SCC compared to normal skin or BCC (**Figure 6**).

### Lack of an Increase of hBD1 and psoriasin Expression in OTR

**Figure 7:**
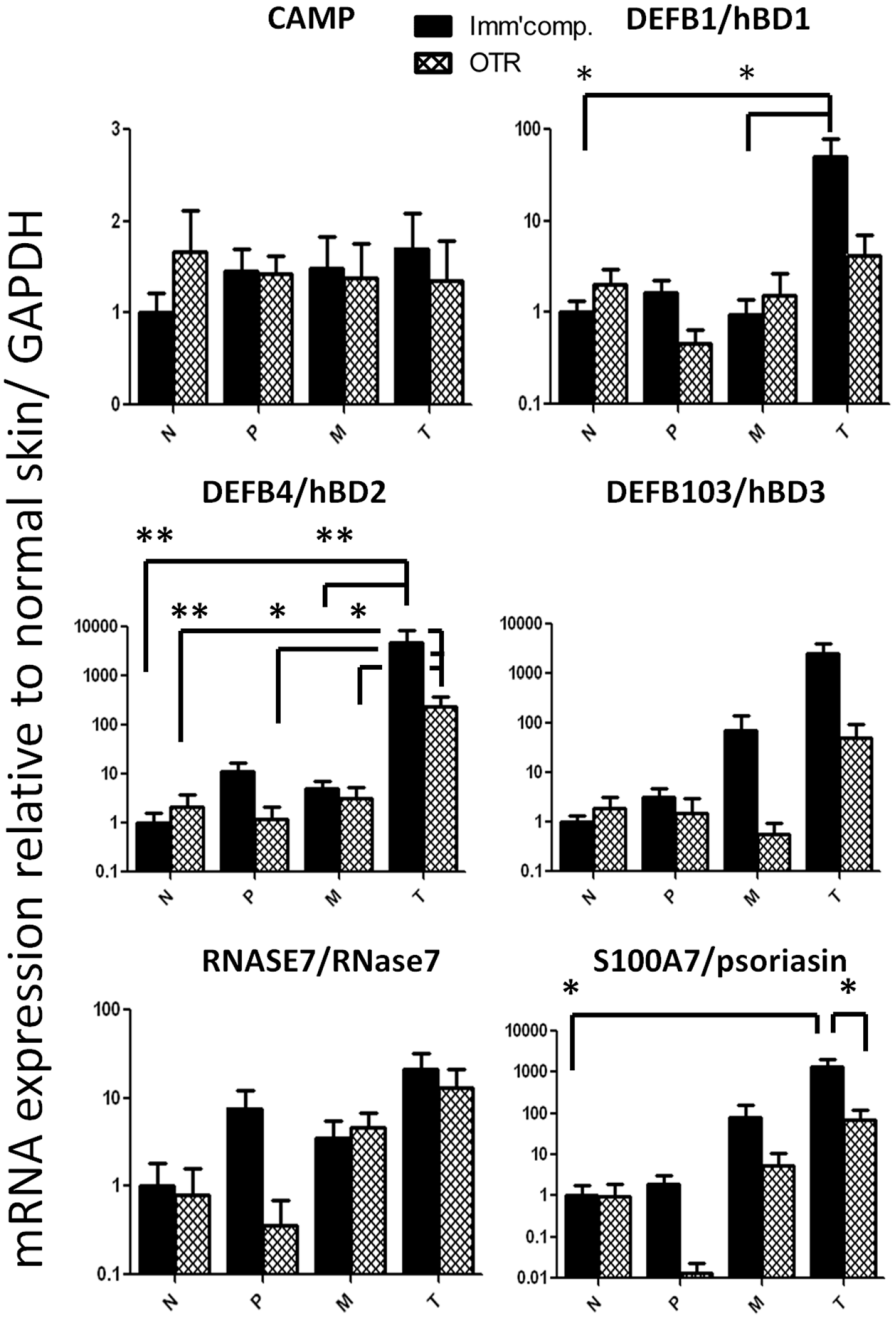
Distinct expression of antimicrobial peptides in squamous cell carcinoma of organ transplant recipients. mRNA expression of cathelicidin antimicrobial peptide (*CAMP*), human β-defensins 1–3 (hBD1-3, genes *DEFB1, DEFB4* and *DEFB103*), RNase 7 (*RNASE7*) and psoriasisin (S100A7) in the tumor center (T), tumor margin (M), peritumoral tissue (P) and distant normal skin (N) in cutaneous squamous cell carcinoma (SCC) of immunocompetent (Imm’comp., black bars) and immunosuppressed patients (OTR  =  organ transplant recipients, cross-hatched bars). Relative mRNA expression to glyceraldehyde 3-phosphate dehydrogenase (GAPDH) and normalized to normal skin is shown. Depicted are mean ± SEM values. *p<0.05, **p<0.01, ***p<0.001 by Kruskal-Wallis test with Dunn’s post-test for comparison between N, P, M and T and by Mann Whitney U test for comparison between immunocompetent and immunosuppressed patients in each group. N = 17 SCC from immunocompetent patients (ICP) and 11 SCC from organ transplant recipients (OTR).

A direct comparison was made between the expression of all target genes in OTR and immunocompetent patients (ICP). Differences in gene expression between OTR and ICP were only found for antimicrobial peptide genes and are displayed in **figure 7**. For all other genes in this study with similar expression between ICP and OTR, both patient categories were merged (n = 28 SCC) for statistical comparison to BCCs and for presentation in figures 2–4.

As in ICPs, SCC in OTRs showed enhanced hBD2 mRNA expression in the tumor center (233.5±149.2) compared to the tumor margin (3.11±2.16, p<0.05), peritumoral tissue (1.22±0.95, p<0.01) and normal skin (2.14±1.61, p<0.05). However, in contrast to ICPs, SCC in OTRs did not overexpress hBD1 or psoriasin. Psoriasin mRNA expression in the tumor center of SCC in OTRs was significantly lower than in ICPs (71.9±49.1 vs. 1334±690, p<0.01) (**Figure 7**). Further supporting these findings were our results from immunohistofluorescence staining showing also lower hBD1 and psoriasin protein expression in SCC from OTRs compared to ICPs (Figure 6).

## Discussion

This study sought to provide an in depth analysis of innate immune gene expression in NMSC. The goal of this work was to further understand how our skin responds to the development of tumors and thus gain essential information that can help guide therapy. The candidate genes evaluated represented those such as TLRs that are involved in the innate immune recognition of danger, cytoplasmic molecules such as TRIF and TRAF that transmit information detected by these receptors, and molecules triggered by this process. These last groups of effector molecules are the AMPs and cytokines responsible for mediating the host response once danger is detected. Results show a dramatic difference in expression of many of these critical molecules in SCC, suggesting that this tumor type has enhanced many elements of innate immune response compared to normal skin. Since many of the effector molecules may play a role in the growth of the tumor or resistance to infection, and some are further altered in the immunocompromised host, these may partially explain the natural progression of NMSC.

Although full coverage of all molecules that participate in innate immunity would involve transcriptional profiling of several thousand gene products, and would be best approached by micro-array analysis [27] or RNASeq [28], the approach of real-time PCR analysis used here is the most quantitative and is commonly used for validation of high-throughput transcriptional profiling results. Sample selection from each patient involved the tumor itself, peritumoral tissue and normal skin. Since each tissue specimen represented a heterogenous collection of cell types, our evaluation is limited by the inability to attribute the mRNA measured to specific resident cells, the malignancy itself, or recruited cell types. However, the data obtained reflected expected expression patterns for the tumor. For example, cutaneous SCC typically retains characteristics of squamous differentiation [29], and we found elevated filaggrin and keratin 10 gene expression in the SCC tissue of the tumor center. Expression was not only higher than in normal skin but also higher than in the tumor margin and in BCC. These results underline substantial differences in differentiation between SCC, BCC and normal skin, and served to validate the samples selected.

Activation of TLRs can result in a wide variety of responses that include apoptosis, inflammatory and non-inflammatory reactions (reviewed in [11]). TLRs are known to be expressed on a variety of murine and human cancer cells (reviewed in [30]). However, little information is available on TLR expression in SCC, and this has come mostly from cervical [31]–[32] or head and neck SCC [33]–[34], but not from cutaneous SCC. Our study showed significantly elevated expression of TLR1,2,3,5,6,7 and 8 in cutaneous SCC. For several TLRs a 5- to 10-fold induction was seen in SCC. Notable responses unique to SCC included TLR5 (responsible for recognition of flagellin), TLR7 (the target of imiquimod), and TLR8, which along with TLR7, can recognize RNA. The TLR8 response was particularly interesting as it also included an induction in the peri-tumoral region. We also observed high TLR2 expression in SCC which could be a characteristic of SCC or a result of a defective skin barrier in SCC. In this way the increase is comparable to the TLR2 upregulation seen in skin wounds [35]. Equally remarkable was the relatively little change in innate immune gene expression seen in BCC. This may be a pertinent negative response given that some TLR induction would be expected from damaged skin. To define a potential functional role of TLR overexpression in cutaneous SCC, further functional studies are needed.

TRIF and TRAF are downstream signaling molecules of TLR activation and their expression correlated with increased TLR expression in SCC. Similarly, the cytokine response was highest in SCC and correlated with increased TLR expression and signaling. However, in BCC TNF was significantly elevated compared to normal skin despite a lesser TLR response. Increased IFNγ was seen only in BCC. This underlines fundamental differences of innate immune receptor signaling and cytokine expression in BCC and SCC and likely reflects both the local expression of these molecules and expression in recruited inflammatory cells.

Because of the action of AMPs to alter cell growth and differentiation [36], their expression may be highly relevant in cancer. This association has been directly demonstrated for cathelicidin, where in mouse tumor models a lack of cathelicidin resulted in much more rapid tumor growth [21]. In the present study we found increased hBD1, hBD2, hBD3 and psoriasin in SCC compared to normal skin, but no increase in cathelicidin. Previously, enhanced hBD2 expression was also reported for oral SCC tumor cells and intratumoral vascular endothelia [29], [37]–[38]. Furthermore, previous studies reported increased hBD3 in *in-situ* and invasive oral SCC, correlating with specific macrophage recruitment to the lesion site [20], [39]. In contrast, the expression of AMPs in BCCs was much different than SCC. We found a significant decrease of hBD1 expression in the invading tumor margin of BCC but not in the tumor center. A decrease in hBD1 was previously reported in BCC [40]. The functional significance of altered hBD expression in cancer is unclear at present. However, treatment of head and neck SCC (HNSCC) cell lines with hBD3 improved resistance against cis-platin, indicating that higher hBD expression may enhance tumor survival [41].

Psoriasin (S100A7) represents a much different type of molecule than the hBDs or cathelicidin and warrents separate discussion. Psoriasin is a much larger protein, is found in much greater abundance in skin, and has different antimicrobial activity (reviewed in [25]). It also does not appear to be expressed by bone marrow derived cells as is the case with many antimicrobial peptides. Rather, psoriasin is expressed primarily in a variety of epithelia. The functions of psoriasin in immunity are thus less clear although it has been associated with resistance to skin surface colonization by *E. coli* [24]. A previous study described elevated psoriasin mRNA expression in *in-situ* and invasive SCC [42], and thus agreed with our results. Psoriasin overexpression has also been reported for other tumors such as breast cancer [43], where persistent expression in invasive tumor areas was associated with poorer prognosis [44]–[46]. Upregulation of psoriasin at transcriptional and protein level was recently also reported for early oral SCC [19]. Psoriasin can bind αvβ6-integrin to promote invasive behavior in oral SCC cells [47]. Therefore, as was discussed for TLRs and their signaling intermediates, the current findings validate the need for further study of the functional relevance of these antimicrobial peptides and proteins.

Immunocompromised patients such as organ transplant recipients (OTRs) have a 60- to 100-fold increased risk for SCC [4], however the exact mechanisms for how immunosuppressive drugs increase the risk for more frequent and more aggressive SCCs are still largely unknown. Long-term immunosuppressive drug treatment in OTR was recently shown to alter the composition of the SCC peritumoral inflammatory infiltrate. Reduced proportions of CD3+, CD8+, and CD14+ (monocytes), FOXP3+ (regulatory T cells) and 123+ (plasmacytoid dendritic cells) cells but more CD138+ plasma cells in SCC of OTR compared to immunocompetent patients have been reported [48]–[49]. Most OTRs are treated with several immunosuppressive drugs simultaneously, making it difficult to elucidate the exact mechanisms how an immunosuppressant contributes to the increased SCC risk.

To investigate if drug-induced immunosuppression altered innate immune gene expression, we included 11 SCC from OTRs for comparison with SCC from immunocompetent patients. All patients in our study were on a treatment regimen with corticosteroids and the mTOR inhibitor rapamycin for at least a year before tumor diagnosis and excision. In epidemiological studies, switching from an immunosuppressive therapy with calcineurin inhibitors to the mTOR-inhibitor sirolimus was effective to reduce skin carcinogenesis in renal transplant patients by 50% [50]. In our study, comparison of expression of all innate immune genes revealed that in contrast to immunocompetent patients, SCC in OTR had lower hBD1 and psoriasin expression, but the abundance of expression of other genes measured was similar to SCC from immunocompetent patients. It would be of interest to determine if the lower expression of hBD1 and psoriasin might be functionally relevant and related to the worse outcome in these patients.

In summary, this study clearly shows that the expression of several genes involved in the innate immune response is different in SCC than normal tissues. There are many possible reasons why skin tumors showed elevated expression of these innate immune genes. Tumor infiltrating immune cells such as CD4^+^, CD8^+^ and FOXP3^+^ T-cells and macrophages secrete soluble effector molecules such as interferons, IL-6 and TNF, and some of these can directly influence innate immune gene expression in keratinocytes (reviewed in [12]). The higher degree of differentiation in SCC compared to BCC or normal basal keratinocytes might be another reason for overexpression of some innate immune genes, since highly differentiated keratinocytes showed enhanced expression of human β-defensins 2 and 3 [51]. A third driver of innate immune gene expression in SCC could be a defective skin barrier in these tumors. Skin SCCs often rupture and form wounds. Previous studies in non-malignant, sterile wounding experiments showed a strong induction of TLR2 and antimicrobial peptides in the wound edge [35]. Furthermore, uncontrolled growth of tumor cells and tissue necrosis lead to release of molecules such as cellular DNA, heat shock proteins and fibrinogen, all of which are danger signals that can activate TLRs and their downstream signaling and effector molecules [52]–[53]. However, even though these mechanisms might be responsible for part of the profile of innate immune gene expression which we found in our study, they provide only an incomplete explanation. Moreover, we believe that expression of some of these innate immune genes is tumor-intrinsic and independent from the tumor environment. Further studies are needed to better understand the exact mechanisms that lead to this distinct innate immune gene expression.

The approach of sampling tissue from the tumor margin and periphery distinguished expression of some genes of interest and provides an example of how basic research might be integrated in well established standardized clinical surgical procedures such as Mohs surgery. Furthermore, it would be a mistake to overlook the remarkably low response seen in BCC for some gene products. The expression (or lack of expression) of unique elements of the innate immune system provides clues to development of the next generation of targeted immune therapy for NMSC.

## Materials and Methods

### Patients

The study was designed to include 10 – 20 specimens each from SCC in immunocompetent patients, from SCC in organ transplant recipients and from basal cell carcinomas. Following approval by the Human Research Protection Program at the University of California, San Diego (IRB ref. # 071032), and upon receiving written informed consent prior to biopsy, skin specimens from subsequent patients undergoing Mohs surgery for non-melanoma skin cancer at the Division of Dermatology, University of California, San Diego, from December 2009 to June 2010 were collected. The only inclusion criterion was the tumor diagnosis. There were no exclusion criteria. None of the patients in this study was pregnant or had other underlying skin disorders in the areas from which skin samples were obtained. Storage and use of all tissues included in the work presented here was carried out in accordance with the Helsinki declaration. 37 patients were included. 19 patients had a basal cell carcinoma (BCC) and 28 squamous cell carcinoma (SCC). Because immunosuppressed patients such as organ transplant recipients (OTR) have a 60–100-fold greater risk for SCCs this study was designed to include 11 SCCs from OTRs for comparison with 17 SCC from immunocompetent patients (ICP). In the BCC patient group, 68.4% were men, and mean age was 67.1±3.9 years (± SEM). In the SCC ICP group, 76.5% were men and mean age was 74.3±2.7 years. In the SCC OTR group, 81.8% were men. As expected and published before [4], [54], OTRs in this study developed SCC significantly earlier than ICPs (at 49.2±2.2 y, p<0.001) (**Figure 1A,B**). Transplanted organs were as follows: 8 kidneys, 1 liver and 2 hearts. Time since transplantation was ranging from 8 to 31 years. The current immunosuppressive regimens for at least one year before Mohs surgery were rapamycin & prednisone.

### Collection of Human Skin Specimens

Mohs microscopically controlled surgery of these skin tumors was performed as described before [55]. During the course of Mohs surgery, 2x2x2 mm^3^ specimens were collected from the center of the tumor (T), from the margin of the tumor (M) consisting of tumor cells and non-tumor cells, from the peritumoral region (P) in close proximity to the tumor but without any tumor cells, and from normal skin (N) in far distance from the tumor, used to reconstruct the skin defect after complete tumor excision (**Figure 1C**). This approach allowed comparison of not only tumor tissue with normal skin in the same patients but also with the peritumoral microenvironment and with the invading tumor edge.

### Quantitative Real-time PCR (qPCR)

Total RNA was isolated using TRIzol reagent (Invitrogen, Carlsbad, CA) and 1 µg RNA was reverse transcribed using iScript (BioRad, Hercules, CA). Expression of the cathelicidin gene (*CAMP*) was evaluated using the FAM-CAGAGGATTGTGACTTCA-MGB probe with primers 5′-CTTCACCAGCCCGTCCTTC-3′ and 5′-CCAGGACGACACAGCAGTCA-3′. Taqman gene expression assays detecting human loricrin (Hs01894962_s1), filaggrin (Hs00856927_g1), keratin 5 (Hs00361185_m1), keratin 10 (Hs00196158_m1), TLR1-9 (Hs00413978_m1, Hs00610101_m1, Hs01551078_m1, Hs00152937_m1, Hs01019558_m1, Hs00271977_s1, Hs00607866_m1, Hs00370913_s1), TRIF (Hs00706140_s1), TRAF1 (Hs01090170_m1) TRAF2 (Hs00184186_m1), TNFα (Hs00174128_m1), IFNγ (Hs00989291_m1), human β-defensins 1-3 (Hs00608345_m1, Hs00823638_m1, Hs00218678_m1), RNAse7 (Hs00261482_m1) and S100A7 (Hs00161488_m1) were purchased from Applied Biosystems (ABI, Foster City, CA). Gene expression was normalized against GAPDH. For GAPDH expression a VIC CATCCATGACCACCCCTGGCCAAG-MGB probe with primers 5′-CTTAGCACCCCTGGCCAAG-3′ and 5′-TGGTCATGAGTCCTTCCACG-3′ was used. RT-qPCR was run on a 7300 Real Time PCR System (ABI). The ΔΔCt method was used to calculate relative expression normalized to normal skin.

### Immunostaining

For immunohistofluorescence, frozen sections (6 µm) were fixed with 4% paraformaldehyde, blocked with 3% BSA in phosphate-buffered saline (PBS), and incubated with mouse monoclonal anti-psoriasin antibody (47C1068, sc-52948, Santa Cruz Biotechnology), rabbit anti-hBD1 (FL-68, sc-20797, Santa Cruz) or anti-hBD2 antibody (FL-64, sc-20798, Santa Cruz). Normal mouse IgG (sc-3877, Santa Cruz) and rabbit pre-immune serum were used as negative controls (**Figure S1**). After washing with PBS, FITC-conjugated goat anti-mouse IgG (Sigma-Aldrich) or FITC-conjugated goat anti-rabbit IgG (Jackson Immuno Research Laboratories, West Grove, PA) was used as second antibody. Sections were mounted in ProLong Gold Anti-Fade reagent with 4′-6-diamidino-2-phenylindole (Molecular Probes/Invitrogen, Eugene, OR). Images were obtained using an Olympus BX41 fluorescent microscope (Scientific Instrument Company, Temecula, CA).

### Statistical Analysis

Statistical analysis was performed using GraphPad Prism software v5.0 (GraphPad Software Inc., La Jolla). The means and standard errors of the mean (SEM) were calculated for each data set. Data were analyzed by analysis of variance (ANOVA) or the Kruskal-Wallis test with post-tests when appropriate, Mann-Whitney U test and unpaired t test. P-values < 0.05 were considered significant.

## Supporting Information

Figure S1 **Negative control staining for hBD1, hBD2 and psoriasin.** Squamous cell carcinomas from immunocompetent patients (SCC imm.comp.) and basal cell carcinomas (BCC) were stained with normal control antibodies. For hBD1 and hBD2 (top row) normal rabbit pre-immune serum was used. For psoriasin (bottom row) normal mouse IgG control antibody was used. FITC-conjugated goat anti-rabbit and goat anti-mouse IgG (green) were used as secondary antibodies. Nuclei were visualized with 4′-6-diamidino-2-phenylindole (DAPI, blue).
(TIF)
