# Supplementary figures and images for: Distinct Innate Immune Gene Expression Profiles in Non-Melanoma Skin Cancer of Immunocompetent and Immunosuppressed Patients
Source: PLoS One. 2012 Jul 13;7(7):e40754. doi: 10.1371/journal.pone.0040754 (PMC3396607; doi:10.1371/journal.pone.0040754)

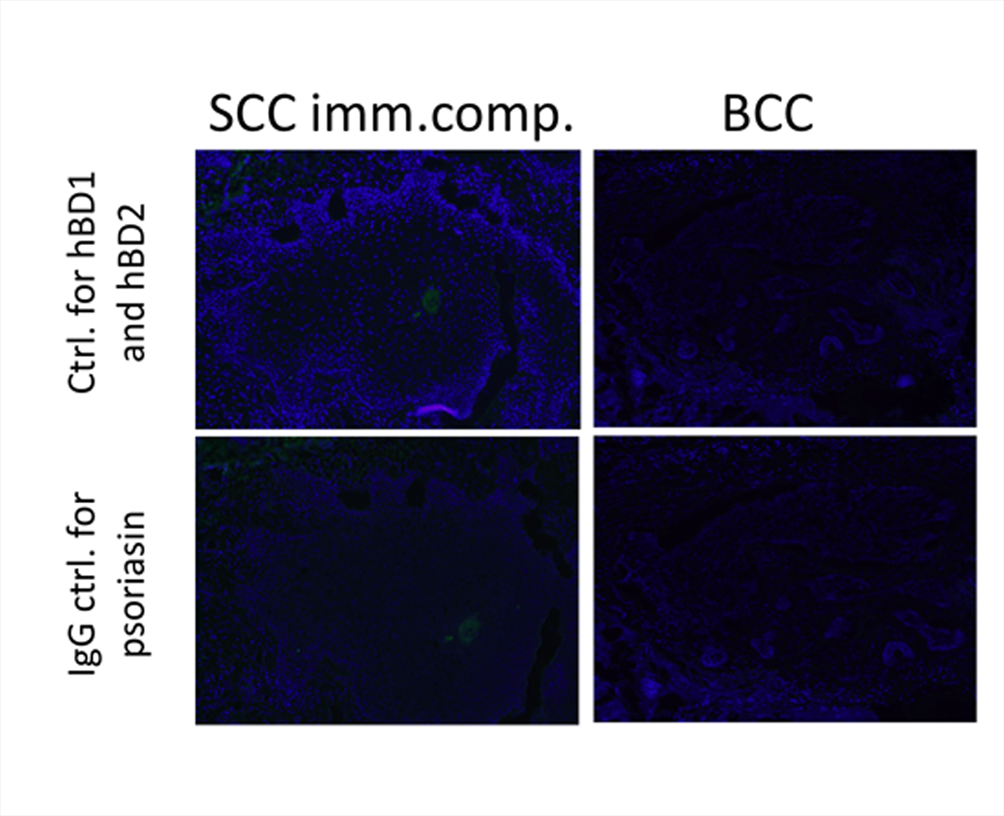

Supplement: Figure S1 — Negative control staining for hBD1, hBD2 and psoriasin. Squamous cell carcinomas from immunocompetent patients (SCC imm.comp.) and basal cell carcinomas (BCC) were stained with normal control antibodies. For hBD1 and hBD2 (top row) normal rabbit pre-immune serum was used. For psoriasin (bottom row) normal mouse IgG control antibody was used. FITC-conjugated goat anti-rabbit and goat anti-mouse IgG (green) were used as secondary antibodies. Nuclei were visualized with 4′-6-diamidino-2-phenylindole (DAPI, blue). (TIF) [file pone.0040754.s001.tif]
